# De novo chronic lymphocytic leukemia/prolymphocytic leukemia or B-cell prolymphocytic leukemia? The importance of integrating clinico-morphological and immunophenotypic findings in distinguishing chronic lymphoproliferative diseases with circulating phase

**DOI:** 10.4322/acr.2020.196

**Published:** 2020-12-08

**Authors:** Zachariah Chowdhury, Yookarin Khonglah, Susmita Sarma, Pranjal Kalita

**Affiliations:** 1 Homi Bhabha Cancer Hospital/MPMMCC (Tata Memorial Hospital), Department of Pathology, Varanasi, Uttar Pradesh, India; 2 North Eastern Indira Gandhi Regional Institute of Health & Medical Sciences, Department of Pathology, Shillong, Meghalaya, India

**Keywords:** Immunophenotyping, Leukemia, Lymphoid, Lymphadenopathy: Rare Diseases

## Abstract

B-cell prolymphocytic leukemia (B-PLL) is an extremely rare disease, accounting for approximately 1% of the lymphocytic leukemias. B-PLL generally occurs in older people. It is characterized by the presence of more than 55% prolymphocytes in the peripheral blood (PB), no or minimal lymphadenopathy, massive splenomegaly, and very high white blood cell counts. The prognosis of B-PLL patients is generally poor, with a median survival of 3 years, although a subset of patients may show a prolonged survival. Herein, we report a case of a 70-year-old male with weakness, generalized lymphadenopathy, and moderate splenomegaly at the initial presentation. Hematologic examination revealed lymphocytic leukocytosis, favoring a chronic lymphoproliferative disorder (CLPD). The key to decoding the precise CLPD was a combination of the clinical profile, morphologic findings on the peripheral blood and the bone marrow, immunophenotypic analysis, and cytogenetic study. The best diagnosis proffered was a de novo chronic lymphocytic leukemia/prolymphocytic leukemia. There was no prior history of lymphoproliferative disorder or lymphocytic leukocytosis. Discriminating this entity from other lymphoproliferative disorders is crucial as the treatment and prognosis are varied compared to the other lymphoproliferative disorders. The diagnostic conundrum encountered and the incredible utility of ancillary studies in such a scenario are highlighted in this study.

## INTRODUCTION

B-cell prolymphocytic leukemia (B-PLL) is a sporadic lymphoproliferative disorder, accounting for < 1% of all the mature B cell malignancies.^1^ It is thought to arise de novo because the progression of chronic lymphocytic leukemia (CLL) into B-PLL does not occur by definition.^2^ It is characterized by neoplastic proliferation of prolymphocytes with prominent splenic involvement. The critical distinction of B-PLL from CLL and Chronic lymphocytic leukemia/Prolymphocytic leukemia (CLL/PLL) is based on the percentage of prolymphocytes in the peripheral blood. Accordingly, the FAB (French American British) group proclaimed the following classification, which is universally accepted:^2^ ^,^ ^3^ (i) Chronic Lymphocytic Leukemia (CLL): when prolymphocytes are ≤ 10% of lymphoid cells; (ii) CLL-PLL: when prolymphocytes are 11-55%; (iii) PLL: when prolymphocytes are > 55% of lymphoid cells.

Other chronic lymphoproliferative disorders (CLPDs) also obfuscate the picture of B-PLL and CLL/PLL. The distinction is crucial given the different therapeutic strategies for the various LPDs. Herein, we report a case of CLPD in this context, and attempt to bring forth the diagnostic perils, and also the pointers to unravel the dilemma.

### Case Report

**Figure 1:**
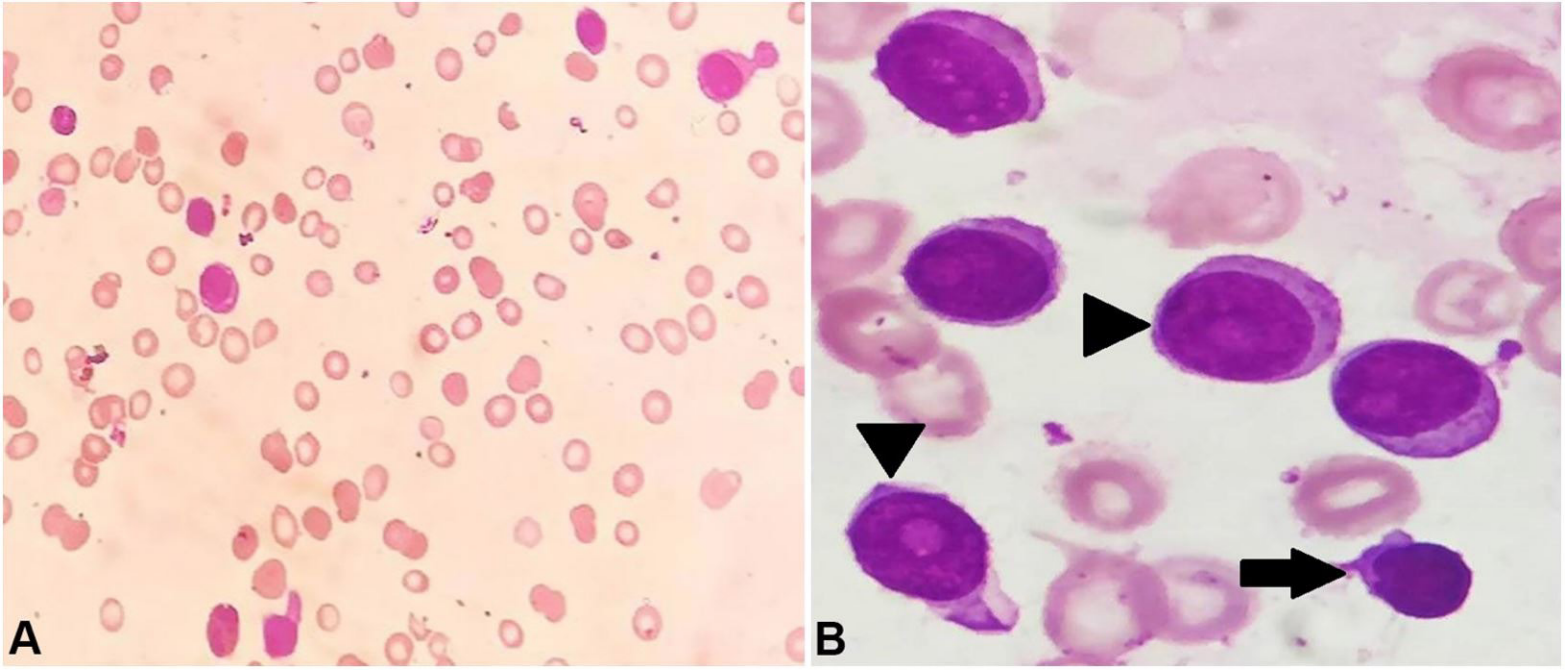
**A –** Photomicrograph of peripheral blood smear showing lymphocytosis comprising atypical lymphoid cells; **B –** Photomicrograph of bone marrow aspirate exhibiting dual population of atypical lymphoid cells – small CLL lymphocyte (arrow) and prolymphocytes (arrowheads) [Leishman stain (A) X400, (B) X1000].

A 70-year-old male presented at our institute with decreased appetite and generalized weakness for the past 4 weeks. He did not complain of fever and weight loss. On examination, multiple bilateral axillary, cervical, inguinal, and left supraclavicular lymphadenopathy (largest measuring 2.5 x 2 cm) were noted, along with moderate splenomegaly. The patient had no prior history or documentation of lymphocytic leukocytosis or any relevant illness. Hematological parameters revealed macrocytic anemia [Hemoglobin: 4.8g/dl (reference range [RR], 12-17g/dl), Mean Corpuscular Volume: 120fl (RR, 80-100fl), Mean Corpuscular Hemoglobin: 40pg (RR, 27-32pg), Mean Corpuscular Hemoglobin Concentration: 33% (RR, 31-35%)], thrombocytopenia [Platelet count: 30,000/μL (RR, 150000-400000/μL) and leukocytosis [Total Leucocyte Count: 21,000/μL (RR, 4000-11000/μL)] with 80% atypical lymphocytes. Analysis of the lymphoid morphology divulged two population of cells, the predominant (70% of lymphoid cells) being small atypical lymphocytes having round nuclei, clumped chromatin and scant cytoplasm, while the other sort (30% of lymphoid cells) constituted medium-sized cells (twice the size of a small lymphocyte) with a round to indented nucleus, moderately condensed nuclear chromatin, prominent central nucleolus and a moderate amount of cytoplasm (Figure 1A). These latter cells were fancied to be prolymphocytes, and a diagnosis of CLPD was suspected. The flow cytometric analysis was advised for confirmation and subtyping. Since the flow cytometric lymphoma panel in our resource-limited setting did not comprise the required armamentarium to conclusively distinguish between the relevant CLPDs in our context, bone marrow aspiration, and especially, a biopsy was sought in the hope that immunohistochemistry (IHC) could provide succor by providing the requisite immunomarkers, although as per the International Workshop on CLL (iwCLL) guidelines a bone marrow aspirate and biopsy are generally not required for the diagnosis of CLL/PLL.. The bone marrow study disclosed a hypercellular marrow diffusely infiltrated by atypical lymphoid cells (83%), suppressing the normal hematopoietic elements. Appraisal of the lymphoid cells unveiled the same morphology discerned in the peripheral blood; however, the differential count was altered. The majority (68%) of these lymphoid cells were the medium-sized cells, while the remaining (32%) comprised the small atypical lymphocytes (Figure 1B). In addition, a megaloblastic picture in the erythroid series was identified, accounting for the macrocytic anemia. The coagulation profile, liver and renal function tests, viral markers (for hepatitis B, hepatitis C, and HIV) and autoimmune profile (ANA and ANCA) were unremarkable. The abdominal ultrasonography confirmed moderate splenomegaly and mild hepatomegaly.

The flow cytometry of the bone marrow aspirate sample was performed on the four-color enabled BD-FACS Caliber Flow cytometer using the standard lyse-wash-stain procedure. Data were analyzed using the CellQuest Pro Software. The abnormal bright CD-45 positive cells exhibiting low to moderate Forward Scatter and low Side scatter were gated (63%).

The gated cells showed moderate intensity for CD 19, CD 20, CD 22 and kappa. A subset of these cells was positive for CD 79a; while CD 3, CD 4, CD 8, CD 10, CD 11c, CD 103, CD 34 and lambda were negative [All antibodies from BD Biosciences, San Jose, CA, USA]. The immunophenotype was consistent with a mature B-cell CLPD (Figure 22B).

**Figure 2:**
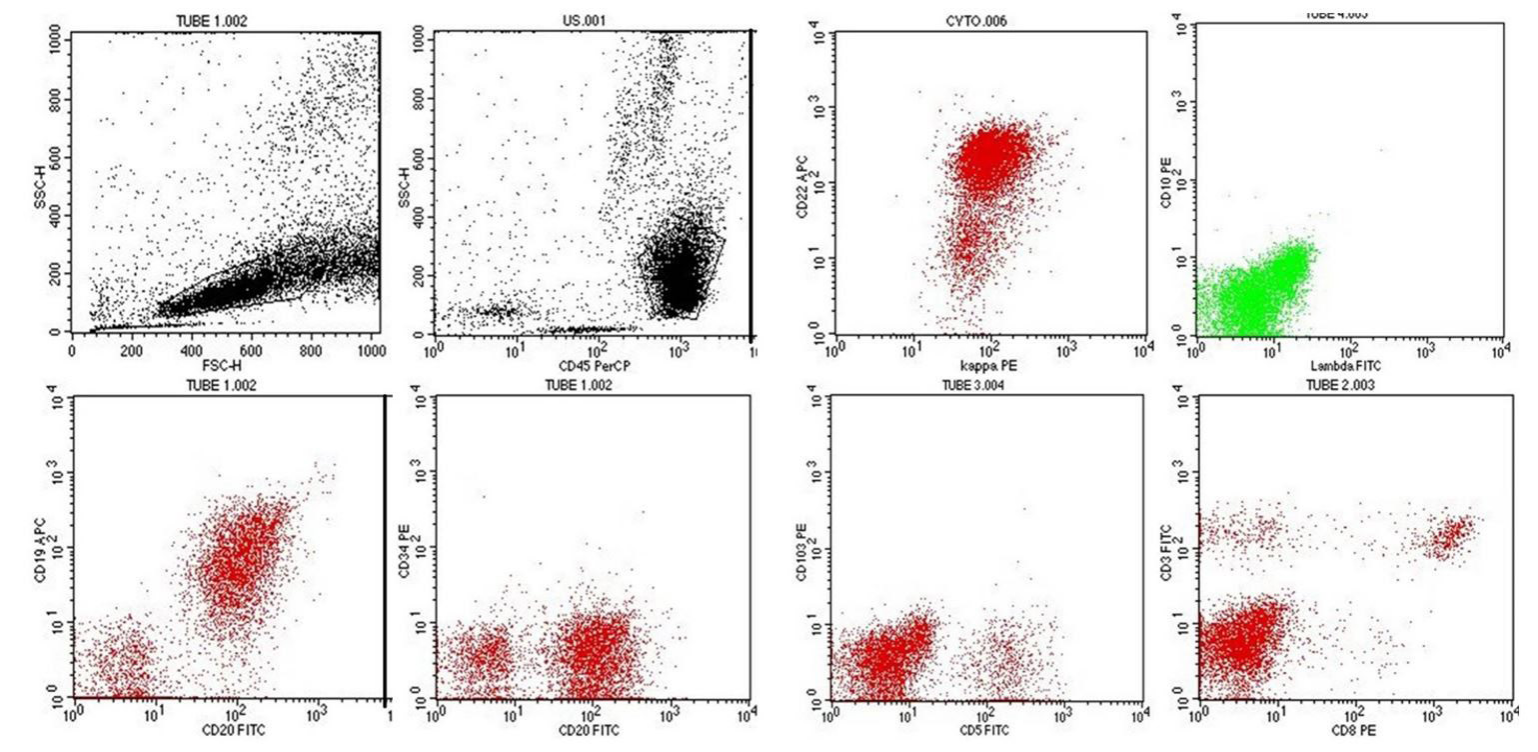
Flow cytometric analysis exhibiting predominance of the lymphoid population which was gated on the CD 45 vs Forward Scatter vs Side Scatter plot. The gated events (63%) demonstrated a CD 19++, CD 20++, CD 22++ clone for light chain kappa and negativity for CD 34, CD 10, CD 103, lambda, surface CD 3, CD 8, CD 4 (not shown) and CD 11c (not shown).

IHC on the bone marrow biopsy demonstrated the atypical lymphoid cells to be negative for Cyclin D1 and positive for CD 5 (Figure 333C). Cyclin D1 was resorted to by IHC on the marrow biopsy in view of the absence of CD 23 in the flow cytometry panel, with respect to mantle cell lymphoma.

**Figure 3:**
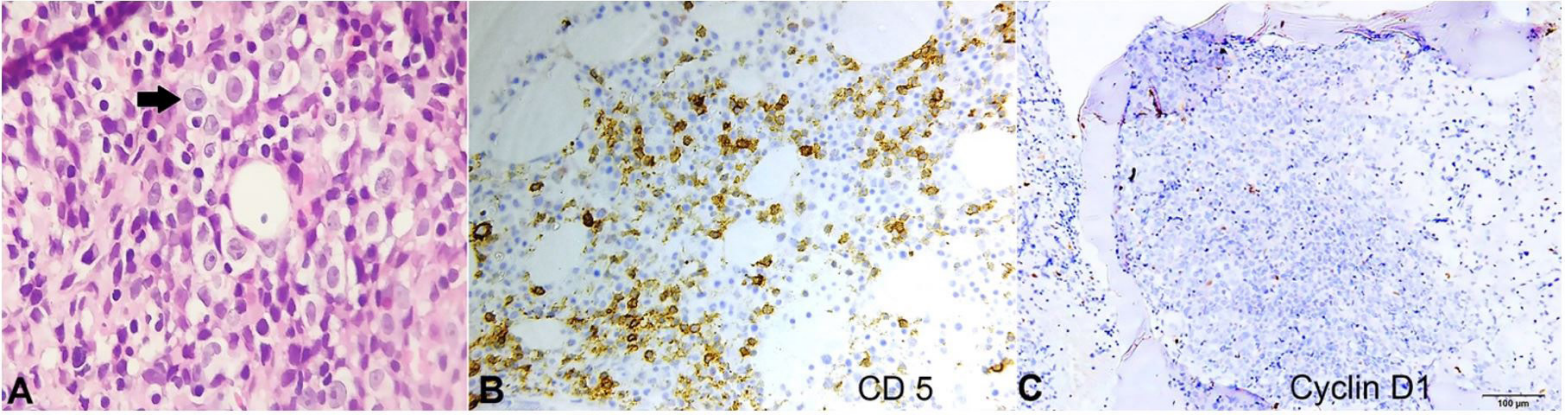
**A –** Photomicrograph of bone marrow biopsy revealing diffuse infiltration by dual population of atypical lymphoid cells, the prolymphocytes (arrow) and the small lymphocytes. IHC demonstrating reactivity of the lymphoid cells for CD 5 in B, and negativity for Cyclin D1 in C (**A –** H & E, X400; **B –** X400; **C –** X100).

Correlating with the clinical findings of generalised lymphadenopathy and moderate splenomegaly (at the first presentation), hematological parameters, morphology and percentage of the abnormal lymphoid cells in the peripheral blood and bone marrow, flow cytometry analysis and IHC study on the marrow biopsy, a final diagnosis of CLL/PLL (de novo) was proffered. Though a conventional karyotyping could not be performed, fluorescence in situ hybridization (FISH) was undertaken for t(11;14), which was negative. The patient was treated with combination chemotherapy with rituximab and bendamustine and was doing well after three cycles of chemotherapy, with normalization of symptoms and blood counts. The patient has been lost to follow-up since then.

## DISCUSSION

B-PLL is an extremely rare, clinically aggressive lymphoid malignancy usually seen in the advanced age (median age of 69 years) with similar incidence in males and females. Most patients present with B symptoms like fever, night sweats, and weight loss, massive splenomegaly without significant lymphadenopathy, and marked lymphocytosis (usually > 100 x 10^9^/L) with numerous prolymphocytes in the peripheral blood (usually > 90%) and bone marrow. Anemia and thrombocytopenia are seen in 50% of cases.^2^ ^,^ ^4^ ^,^ ^5^ Galton et al.^6^ described a prolymphocyte as a large cell with a round nucleus, a prominent vesicular nucleolus, condensed nuclear chromatin, and abundant cytoplasm. The French-American-British (FAB) group subsequently delineated B-PLL as having more than 55% prolymphocytes of the lymphoid cells in the peripheral blood.^4^ In contrast, CLL is a common, clinically indolent neoplasm in which the neoplastic cells are small lymphocytes with mature chromatin and minimal cytoplasm without nucleoli. However, a subset of CLL cases may acquire an increased number of prolymphocytes and eventually transform into a neoplasm that can resemble those of B-cell PLL. These neoplasms have been designated as CLL in prolymphocytoid transformation or CLL/PLL.^2^ ^,^ ^7^ ^,^ ^8^ Both CLL/PL and PLL are characterized by marked splenomegaly, and in both, the extent of splenic enlargement is proportional to the percentage of prolymphocytes. However, there is a striking discontinuity between these two groups, because lymph-node enlargement is a major feature of CLL/PLL but not of PLL. Thus, PLL cannot be considered as the extreme end of a continuous spectrum from typical CLL.^7^ Gene expression profiling has demonstrated that B-PLL has a signature quite distinct from that of CLL or CLL/PLL^9^ and are biologically distinct diseases.

The diagnosis of CLL/PLL is often challenging because of the considerable overlap with other mature B-cell leukemias and lymphomas (Table 1). The differential diagnosis in our context included: (A) B-cell type: (i) CLL, (ii) B-PLL, (iii) Hairy cell Leukemia (HCL), (iv) HCL Variant (HCL-V), (v) Lymphoma spillover: Splenic Marginal Zone Lymphoma (SMZL), Mantle Cell Lymphoma (MCL), and Follicular cell lymphoma. (B) T-cell type: T-cell PLL. The clinical finding of significant generalized lymphadenopathy, as seen in our case, is extremely uncommon in B-PLL, SMZL, HCL, and HCL-V, which are otherwise the closest mimickers given the similar clinical presentation (older age, splenomegaly, and lymphocytosis). Thus, the aforementioned clue is the most substantial one in somewhat excluding these entities. Meticulous scrutiny of peripheral blood morphology is one of the keys to an accurate diagnosis. The prolymphocyte count must be greater than 55% of the lymphoid cells in the peripheral blood and usually exceeds 90% in B-PLL, while in CLL it is less than 11% and in CLL/PLL, 11-55%.^2^ ^,^ ^6^ ^,^ ^7^ It must be firmly borne in mind that the computation has to be performed in the peripheral blood and not in the bone marrow. Thus, while the count of prolymphocytes in our case exceeded 55% in the marrow, but owing to its enumeration being 30% of the lymphoid cells in the peripheral blood, both B-PLL and CLL were excluded morphologically. Moreso, B-PLL presents with a very high total leukocyte count with marked lymphocytosis. The B-prolymphocyte has a characteristic large size, twice that of a small CLL lymphocyte. The nuclear chromatin is moderately condensed, there is often a prominent central nucleolus, and the nuclear outline is typically round and more uniform than in CLL. The cytoplasm is more abundant than in CLL, clear, and only weakly basophilic. The cells of HCL are known to have hairy cytoplasmic projections and uniformly lack nucleoli, unlike that of prolymphocytes. Peripheral blood atypical lymphocyte morphology in SMZL consists of villous lymphocytes (polar villi) with basophilic cytoplasm. Franco et al.^10^ reported that bone marrow infiltration of the SMZL is mostly of the intrasinusoidal type. The closest morphologic differential of prolymphocytes is the atypical lymphoid cells of HCL-V, which has intermediate properties between HCL and B-PLL. However, in contrast to the circulating cells in HCL-V and SMZL, the cytoplasm of prolymphocytes generally has a smooth outline.^3^ ^,^ ^4^ Follicular lymphoma with circulating disease can be delimited by the cleaved nuclei with irregular contours characteristic of centrocytes.^3^

**Table 1:** Differentiating features (Clinical & Immunophenotypic) of the mature B cell lymphomas in the context of our study

**Parameters**	**CLL/PLL**		**B-PLL**	**CLL**	**HCL**	**HCL-V**	**SMZL**	**MCL**	**FL**
Generalised Lymphadenopathy	Present		Absent	Present	Uncommon	Uncommon	Uncommon	Present	Present
Splenomegaly	Moderate to massive		Massive	Mild to moderate	Massive	Moderate to massive	Moderate to massive	Mild to moderate	Mild to moderate
Lymphocyte count	Variably increased		High, > 100 x 10^9^/L	Variably increased	Normal or low, Pancytopenia with monocytopenia	Modest, 20-40 x 10^9^/L; No monocytopenia	Usually normal or low level increase	Usually < 50 x 10^9^/L	Normal or low level increase
Lymphoid cell morphology	Mixture of small CLL lymphocytes and Prolymphocytes (11-55%)		Predominantly prolymphocytes (> 55%), usually > 90%	Predominantly small lymphocytes; prolymphocytes < 11%	Lack nucleoli, nuclei indented, ‘hairy’ cytoplasmic projections	Prominent central nucleolus, irregular nuclear contours, ‘hairy’ cytoplasmic projections	Short polar villi, basophilic cytoplasm	Heterogeneous, larger indented nuclei	Inconspicuous nucleoli, small irregular cleaved nuclei resembling centrocyte
**Immunophenotype**									
B cell antigens (CD 19, CD 20, CD 22, CD 79a)		+ (Moderate)	+ (Strong)	+ (Dim)	+ (Strong)	+ (Strong)	+ (Moderate)	+ (Moderate)	+ (Moderate)
CD 5		+/-	- (most)	+	-	-	- (usually)	+ (most)	-
CD 23		+/-	-	+	-	-	30% + (weak)	-	+/-
Other antigens		CD 10 -	CD 10 -	CD 10 -	CD 11c +, CD 123 +, CD 25 +, CD 103 +, Annexin A1 +	CD 11c +, CD 103 +, CD 25 -, CD 123 -, Annexin A1 -	CD 103 -, CD 123 -, Annexin A1 -, Cyclin D1 -	Cyclin D1 +, SOX11 +	CD 10 +

B-PLL: B cell prolymphocytic leukemia; CLL: Chronic lymphocytic leukemia; CLL/PLL: Chronic lymphocytic leukemia/prolymphocytic leukemia; FL: Follicular lymphoma; HCL: Hairy cell leukemia; HCL-V: Hairy cell leukemia-variant; MCL: Mantle cell lymphoma; SMZL: Splenic marginal zone lymphoma.

Although immunophenotyping may support a diagnosis of CLL/PLL, a CLL/PLL-specific immunophenotype has not been identified yet;^11^ thus, the diagnosis rests mainly on the exclusion of other conditions. The cells in CLL/PLL express various pan B-cell antigens with moderate intensity (CD 19, CD 20, CD 22, CD 24, CD 79b and FMC 7), and surface immunoglobulin (IgM or IgM/IgD) is detected at higher levels than in CLL.^4^ CD 200 is weakly positive or negative. The higher intensity of the expressed markers helps in differentiating CLL/PLL from CLL, which generally has a dim expression of surface Ig, CD 20, and other B-cell antigens.^1^ ^,^ ^4^ ^,^ ^12^ CLL/PLL can be CD 23 and CD 5 negative; the CD 5 positive cases (as in our context demonstrated by IHC), however, may be difficult to differentiate from MCL in the leukemic phase.^4^ ^,^ ^12^ Herein comes the role of Cyclin D1, which was demonstrated to be negative on the marrow biopsy by IHC. Moreover, cytogenetic analysis of our case did not divulge t(11;14), thus essentially ruling out one important mimicker, i.e., MCL. Also, the absence of expression of CD 11c, CD 103, and CD 10 assisted in distinguishing CLL/PLL from HCL, HCL-V, and follicular lymphoma. The above-mentioned phenotype readily precludes T-PLL, which expectedly expresses T-cell markers. The diagnosis of de novo CLL/PLL was tendered since the patient did not have any prior documentation of lymphocytosis or lymphadenopathy or history of CLL. The presentation of generalized lymphadenopathy with a mixed hematological picture of small lymphocytes and prolymphocytes (30% of lymphoid cells) in the peripheral blood, as well as in the bone marrow fitted into none of the aforementioned entities other than CLL/PLL.

There is a scarcity of data to guide therapy in CLL/PLL cases.^13^ The regimens used for CLL are often employed for treating CLL/PLL. Extrapolating from the CLL guidelines, 17p deletion or TP53 mutation is considered a high-risk genetic feature and is often used to guide therapy. Patients lacking a 17p deletion or TP53 mutation are initially treated with a combination of fludarabine, cyclophosphamide, and rituximab. Conversely, patients with a 17p deletion or TP53 mutations inherit primary resistance to purine analog/alkylator-based therapy, thus making the aforementioned chemotherapeutic agents less effective. Case reports have described the response of such patients treated with targeted drugs including ibrutinib, alemtuzumab, idelalisib, and venetoclax.^1^ ^,^ ^4^ ^,^ ^13^ ^,^ ^14^ Although such regimens with superior efficacy are existent, the patient in our study was treated with bendamustine-rituximab, in view of the financial constraints.

In conclusion, a comprehensive approach involving clinical, morphological, and immunophenotypic features is extremely vital in this era for diagnosis, management, and prognostication. This case, the first report of this entity from North East India, documents the rare de novo presentation of CLL/PLL and also illustrates the impressive utility of ancillary studies such as immunophenotyping as an adjunct to morphology in the diagnosis of this entity and its distinction from other CLPDs.
